# Equity in early detection: the power of communication in bridging the breast cancer care gap in the Americas

**DOI:** 10.1016/j.lana.2024.100771

**Published:** 2024-05-09

**Authors:** The Lancet Regional Health – Americas

Breast cancer has the highest incidence among all cancers, both globally and in the Americas, where 2,822,722 diagnoses and 109,428 deaths from breast cancer were recorded in 2022. Current estimates predict that cases will increase 25% in North America and 50% in Latin America and the Caribbean (LAC) by 2045. Timely early detection and access to treatment are crucial steps in improving outcomes and saving the lives of people with breast cancer. However, people with low incomes and those from minoritised populations are often diagnosed at later stages and face greater barriers to treatment access, increasing their risk of dying from breast cancer.

“Early detection saves lives” is a mantra echoed by health-care professionals, researchers, and civil society advocates. Indeed, all countries that successfully reduced their breast cancer mortality rates in the last 40 years did so by increasing early diagnosis rates and ensuring that at least 60% of invasive breast cancers were diagnosed at stages I or II. Such achievement is referred to as stage-shifting, which can be achieved with increased awareness of early detection importance. However, access to screening mammography and timely diagnostic services remain elusive for many individuals. Moreover, many people do not know the suggested guidelines for screening. They are unaware of the recommended age of first screening, the interval between screenings, and even where to go for their tests.

In October 2023, the International Agency for Research on Cancer and the Pan American Health Organization launched the first edition of the LAC Code Against Cancer. Following the framework of the European code, the LAC code compiled 17 recommendations that individuals can adopt to reduce their risk of developing or dying from cancer. In a linked comment, Maria Constanza Camargo and colleagues defend that the dissemination of such code—published in English, Spanish, and Portuguese and targeted to the lay audience and policymakers—can significantly impact awareness and education about the importance of breast cancer screening and early detection, provided that multisectoral efforts towards implementation are established.

Evidence shows that even brief interventions have the potential to increase women's breast cancer awareness, yet is unlikely that we will advance on equity in early detection without the implementation of sustained and targeted outreach and education initiatives. Partnerships between community organisations and health-care providers have shown success in overcoming access and communication. A small implementation study in a rural Mexican community showed the potential of a school-based awareness programme to achieve intergenerational impact by improving students’ and direct family members’ knowledge of breast health. In the USA, Washington DC has the greatest disparities in incidence and mortality from breast cancer in non-Hispanic Black women compared to non-Hispanic White women. A multi-level community-based pilot programme implemented in an under-resourced urban area of primarily non-Hispanic Black women and Hispanic women of the city was able to increase genetic counselling for risk assessment for hereditary breast and ovarian cancer syndrome from 13% to almost 99%. By direct engaging with systematically marginalised and stigmatised populations, health-care providers can dispel myths surrounding breast cancer screening and provide culturally competent education about the importance of early detection. When it comes to genetic counselling and risk assessment, a culturally sensitive narrative is key to intervention success.

Health literacy—or the lack of it—permeates several of the neglected areas in breast cancer care, from engagement in screening and genetic assessments to adherence to treatment and understanding the outcomes and expectations. Although efforts to increase awareness, such as the recently released LAC Code Against Cancer, are commendable and essential, we highlight the importance of a patient-centred approach that goes beyond a list of dos and don’ts, honouring the patient’s capacity to understand their body's functioning and empower them through knowledge to participate in the decision plan around their health.

The importance of improved communication between health-care providers and patients is a key call for action in the recently published *Lancet* Breast Cancer Commission, and we echo the recommendation that 100% of health-care workers must receive skills training in person-centred communication. On wider reflection, countries deeply affected by historical racism, like those of the Americas region, should acknowledge the urgency in training health-care professionals to conduct emphatic conversations that are respectful of the patient’s beliefs and honour their personal decisions. Recognising the historical context where specific populations, including Black and Indigenous people, have been systematically excluded from decision-making processes, it seems exceptionally important for our region to embrace the importance of patient involvement in all levels of breast cancer care.

We urge health-care workers, policymakers, and civil advocates in the Americas to embrace the call for innovative and emphatic approaches to breast cancer care, focusing on participatory and empowering approaches to bridge the equity gap in breast cancer screening, achieve greater than 60% early-stage diagnosis, and save millions of lives. In the realm of breast cancer care, achieving equity is not just a noble aspiration but an urgent imperative.

